# Violet light suppresses lens-induced myopia via neuropsin (OPN5) in mice

**DOI:** 10.1073/pnas.2018840118

**Published:** 2021-05-24

**Authors:** Xiaoyan Jiang, Machelle T. Pardue, Kiwako Mori, Shin-ichi Ikeda, Hidemasa Torii, Shane D’Souza, Richard A. Lang, Toshihide Kurihara, Kazuo Tsubota

**Affiliations:** ^a^Department of Ophthalmology, Keio University School of Medicine, Shinjuku-ku, 160-8582 Tokyo, Japan;; ^b^Laboratory of Photobiology, Keio University School of Medicine, Shinjuku-ku, 160-8582 Tokyo, Japan;; ^c^Center for Visual and Neurocognitive Rehabilitation, Atlanta Veterans Affairs Healthcare System, Atlanta, GA 30033;; ^d^Biomedical Engineering, Georgia Institute of Technology and Emory University, Atlanta, GA 30322;; ^e^Visual Systems Group, Abrahamson Pediatric Eye Institute, Division of Pediatric Ophthalmology, Cincinnati Children’s Hospital Medical Center, Cincinnati, OH 45229;; ^f^Department of Ophthalmology, College of Medicine, University of Cincinnati, Cincinnati, OH 45221;; ^g^Tsubota Laboratory, Inc., 160-0016 Tokyo, Japan

**Keywords:** neuropsin (OPN5), myopia, violet light, nonvisual photoreceptors

## Abstract

Myopia has become a major public health concern, particularly across much of Asia. It has been shown in multiple studies that outdoor activity has a protective effect on myopia. Recent reports have shown that short-wavelength visible violet light is the component of sunlight that appears to play an important role in preventing myopia progression in mice, chicks, and humans. The mechanism underlying this effect has not been understood. Here, we show that violet light prevents lens defocus–induced myopia in mice. This violet light effect was dependent on both time of day and retinal expression of the violet light sensitive atypical opsin, neuropsin (OPN5). These findings identify *Opn5*-expressing retinal ganglion cells as crucial for emmetropization in mice and suggest a strategy for myopia prevention in humans.

## Significance

The increasing prevalence of myopia is a significant public health concern. Unfortunately, the mechanisms driving myopia remain elusive, limiting effective treatment options. This report identifies a refractive development pathway that requires *Opn5*-expressing retinal ganglion cells (RGCs). Stimulation of *Opn5* RGCs with short-wavelength violet light prevented experimental myopia in mice. Furthermore, this effect was dependent on the time of day, with evening exposure being sufficient to protect against experimental myopia. Thus, these studies suggest *Opn5* RGCs may contribute to the mechanisms of emmetropization and identify the OPN5 pathway as a potential target for the treatment of myopia.

Myopia (nearsightedness) in school-age children is generally axial myopia, which is the consequence of elongation of the eyeball along the visual axis. This shape change results in blurred vision but can also lead to severe complications including cataract, retinal detachment, myopic choroidal neovascularization, glaucoma, and even blindness (1–3). Despite the current worldwide pandemic of myopia, the mechanism of myopia onset is still not understood (4–8). One hypothesis that has earned a current consensus is the suggestion that a change in the lighting environment of modern society is the cause of myopia (9, 10). Consistent with this, outdoor activity has a protective effect on myopia development (9, 11, 12), though the main reason for this effect is still under debate (7, 12, 13). One explanation is that bright outdoor light can promote the synthesis and release of dopamine in the eye, a myopia-protective neuromodulator (14–16). Another suggestion is that the distinct wavelength composition of sunlight compared with fluorescent or LED (light-emitting diode) artificial lighting may influence myopia progression (9, 10). Animal studies have shown that different wavelengths of light can affect the development of myopia independent of intensity (17, 18). The effects appear to be distinct in different species: for chicks and guinea pigs, blue light showed a protective effect on experimentally induced myopia, while red light had the opposite effect (18–22). For tree shrews and rhesus monkeys, red light is protective, and blue light causes dysregulation of eye growth (23–25).

It has been shown that visible violet light (VL) has a protective effect on myopia development in mice, in chick, and in human (10, 26, 27). According to Commission Internationale de l’Eclairage (International Commission on Illumination), VL has the shortest wavelength of visible light (360 to 400 nm). These wavelengths are abundant in outside sunlight but can only rarely be detected inside buildings. This is because the ultraviolet (UV)-protective coating on windows blocks all light below 400 nm and because almost no VL is emitted by artificial light sources (10). Thus, we hypothesized that the lack of VL in modern society is one reason for the myopia boom (9, 10, 26).

In this study, we combine a newly developed lens-induced myopia (LIM) model with genetic manipulations to investigate myopia pathways in mice (28, 29). Our data confirm (10, 26) that visible VL is protective but further show that delivery of VL only in the evening is sufficient for the protective effect. In addition, we show that the protective effect of VL on myopia induction requires OPN5 (neuropsin) within the retina. The absence of retinal *Opn5* prevents lens-induced, VL-dependent thickening of the choroid, a response thought to play a key role in adjusting the size of the eyeball in both human and animal myopia models (30–33). This report thus identifies a cell type, the *Opn5* retinal ganglion cell (RGC), as playing a key role in emmetropization. The requirement for OPN5 also explains why VL has a protective effect on myopia development.

## Results

### VL at Dusk Suppresses LIM in Mice.

**Fig. 1.**
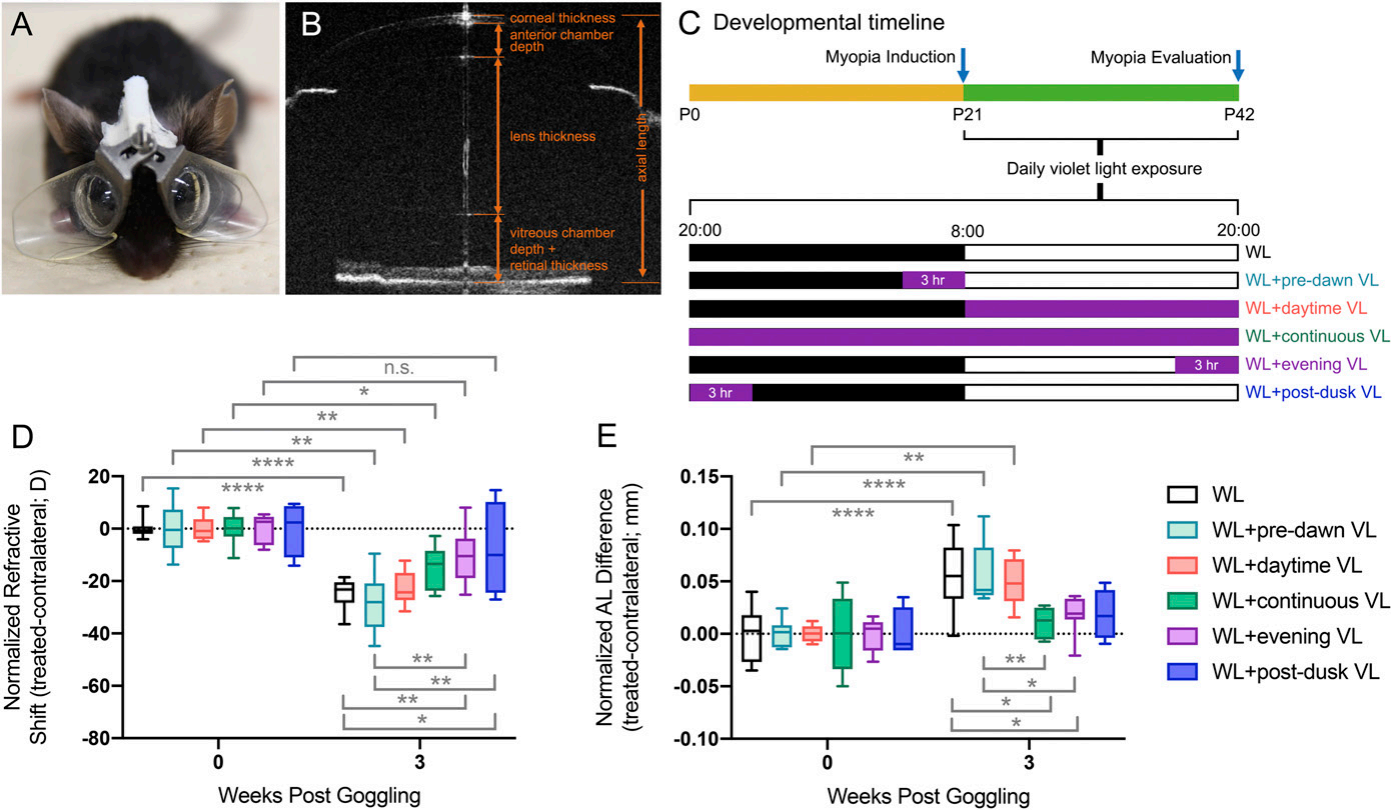
Time of day–specific VL-suppressed LIM progression. (*A*) Mouse with lenses for inducing LIM. In all the experiments using LIM mice, 0-D lenses were attached over left eyes and −30-D lenses were attached over right eyes. (*B*) OCT image of the whole mouse eye showing the different axial measurements, including AL. (*C*) Together with LIM, VL exposure at different times of day was added with white background light from p21 to p42 in each group. The relative refraction difference between eyes in each group with VL exposure at different times of day is shown in *D*, and the relative AL difference between eyes was shown in *E*. The data are displayed as box plots showing the minimum and maximum (error bars,) the interquartile range (box), and the median value (bold horizontal line within the box). The VL exposure for each group was as follows: control group with only WL; VL from 05:00 to 08:00 (WL + predawn VL); VL from 08:00 to 20:00 (WL + daytime VL); continuous VL (WL + continuous VL); VL from 17:00 to 20:00 (WL + evening VL); VL from 20:00 to 23:00 (WL + postdusk VL). *n* = 4 for each group. n.s.: not significant, *P < 0.05, **P < 0.01, ****P < 0.0001. The values and statics for *D* and *E* are shown in *SI Appendix*, Tables S1 and S2, respectively.

To investigate the effect of VL on experimental myopia, we used a newly developed LIM mouse model described elsewhere (28, 29). Briefly, 0 diopter (D) lenses were attached in front of left eyes as internal controls, and −30 D lenses were attached in front of right eyes to induce myopia (Fig. 1*A*). We defined axial length (AL) as the distance from the corneal vertex to the retinal pigment epithelium (RPE) layer near the optic nerve (Fig. 1*B*). The schedule of VL exposure is shown in Fig. 1*C*. Together with the initiation of LIM, mice were exposed to 400 μw/cm^2^ (8.0 × 10^14^ photons/cm^2^/sec, ∼1% of sunlight at this wavelength) of VL from postnatal day 21 (P21). Mice were divided into five groups and exposed to VL at different times each day in addition to the standard mouse room fluorescent lighting (Fig. 1*C* and Table 1). VL exposure protocols included 3 h predawn (Fig. 1*C*, white light [WL] + predawn VL), all day exposure (Fig. 1*C*, WL + daytime VL), continuous VL (Fig. 1*C*, WL + continuous VL), 3 h of evening VL (Fig. 1*C*, WL + evening VL), and 3 h of postdusk VL (Fig. 1*C*, WL + postdusk VL). When these protocols were complete at P42, the relative difference in refraction and AL between right and left eyes normalized to baseline was determined (Fig. 1 *D* and *E* and *SI Appendix*, Tables S1 and S2).

**Table 1.** The summary of grouping and light exposure in each experiment

Figure	Experimental description	Group names (sample size)	Light exposure
1	VL exposure at different times	WL (*n* = 10)	WL only 08:00 to 20:00
		WL + predawn VL (*n* = 7)	WL 08:00 to 20:00
			VL 05:00 to 08:00
		WL + daytime VL (*n* = 5)	WL 08:00 to 20:00
			VL 08:00 to 20:00
		WL + continuous VL (*n* = 6)	WL 08:00 to 20:00
			VL 00:00 to 24:00
		WL + evening VL (*n* = 7)	WL 08:00 to 20:00
			VL 17:00 to 20:00
		WL+ postdusk VL (*n* = 4)	WL 08:00 to 20:00
			VL 20:00 to 23:00
2	Different wavelengths on LIM	WL group (*n* = 8)	WL only 08:00 to 20:00
		WL + RL (*n* = 8	WL 08:00 to 20:00
			RL 17:00 to 20:00
		WL + GL (*n* = 8)	WL 08:00 to 20:00
			GL 17:00 to 20:00
		WL + BL (*n* = 8)	WL 08:00 to 20:00
			BL 17:00 to 20:00
		WL + VL (*n* = 8)	WL 08:00 to 20:00
			VL 17:00 to 20:00
4	Retinal Opn5 contribution to VL effects	Opn5 WT (*n* = 5)	WL only 08:00 to 20:00
		Opn5 WT + VL (*n* = 4)	WL 08:00 to 20:00
			VL 17:00 to 20:00
		Opn5 KO (*n* = 5)	WL only 08:00 to 20:00
		Opn5 KO + VL (*n* = 4)	WL 08:00 to 20:00
			VL 17:00 to 20:00

In this assay, the standard fluorescent lighting control resulted in the expected (28) lens defocus–induced change in refraction and AL (Fig. 1 *D* and *E*, black and white bars). Neither predawn VL (Fig. 1 *D* and *E*, aqua) nor daytime VL (Fig. 1 *D* and *E*, red) had any significant influence on lens-induced refractive shift or change in AL compared to the WL group. By contrast, all other VL exposure protocols produced significant changes in at least one parameter (Fig. 1 *D* and *E*, green, purple, and blue). WL combined with continuous VL (Fig. 1 *D* and *E*, green) significantly suppressed the lens-induced change in AL compared to WL only (Fig. 1*E*, green). WL combined with evening VL (Fig. 1 *D* and *E*, purple) significantly suppressed the degree of lens-induced refractive shift (Fig. 1*D*, purple) and the AL change (Fig. 1*E*, purple) compared to WL only. Finally, 3 h of postdusk VL suppressed the lens-induced changes in refraction (Fig. 1*D*, blue). These results suggested that 3 h of VL exposure just prior to or just after dusk was sufficient to prevent myopia progression in mice. Based on these data, all subsequent light exposure experiments use 3 h of predusk, evening light exposure.

### VL Is the Most Effective Wavelength for Myopia Suppression.

**Fig. 2.**
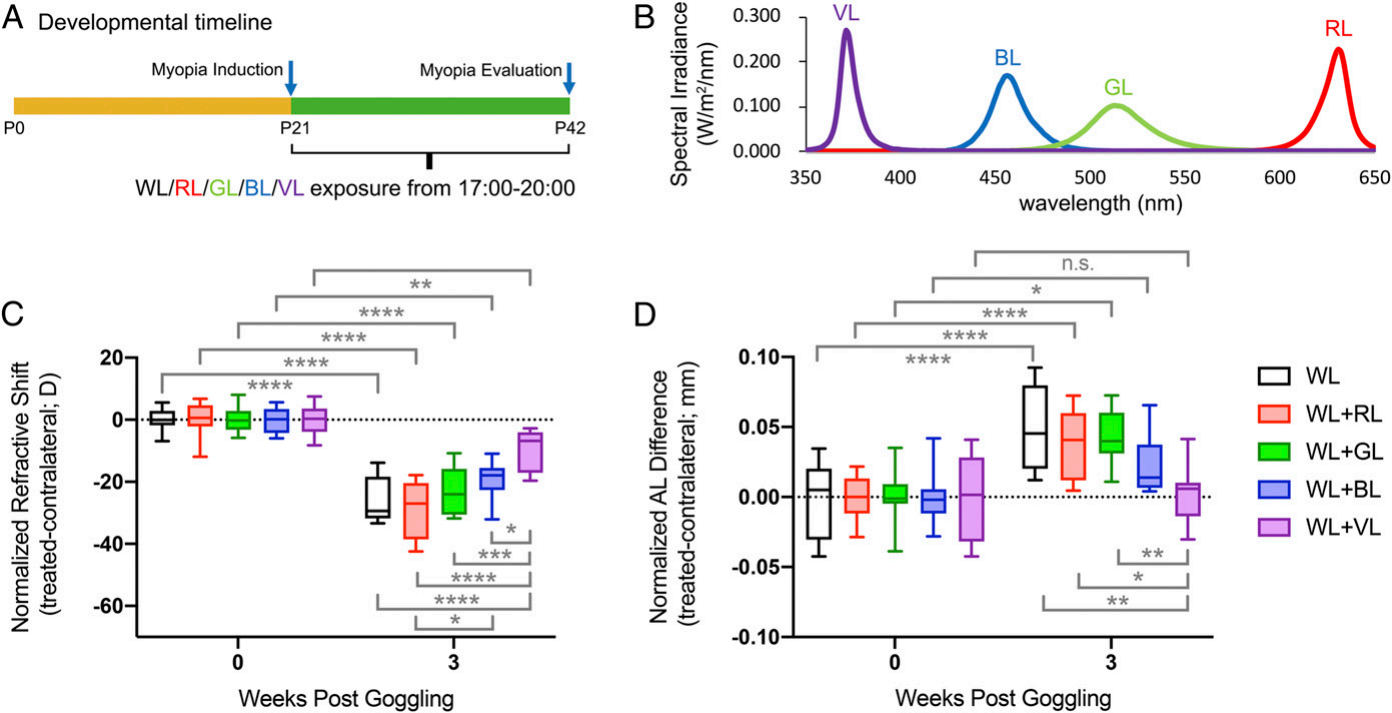
VL was the most effective wavelength for suppressing LIM progression. (*A*) Red, green, blue, and VL exposure was added to the white background light from 17:00 to 20:00 every day (evening) in each group. The control group was exposed to the white background light only. LIM was initiated at P21 and lasted for 3 wk. (*B*) Red, green, blue, and VL had the same irradiance. The relative difference between eyes, normalized to baseline for refractive errors (*C*) and to ALs (*D*) indicates the largest protective effects with VL. The data are displayed as box plots showing the minimum and maximum (error bars), the interquartile range (box), and the median value (bold horizontal line within the box). n.s.: not significant, *P < 0.05, **P < 0.01, ****P < 0.0001. RL: red light; GL: green light; BL: blue light. The values and statics for *C* and *D* are shown in *SI Appendix*, Tables S3 and S4, respectively.

To investigate the wavelength specificity of light in suppression of LIM progression in mice, we compared the effect of VL with blue (440 to 480 nm), green (500 to 540 nm), and red (610 to 650 nm) light. In this protocol (Fig. 2*A*), violet, blue, green, and red light were each adjusted to the same irradiance (400 μw/cm^2^, Fig. 2*B*) and added to white fluorescent light from 17:00 to 20:00 every day (as in Fig. 1, WL + evening VL). Control mice were exposed to WL only (*n* = 8 in each group). At P42, we measured the relative refractive shift and the AL between eyes with control, 0-D lenses, and experimental −30-D lenses normalized to baseline. Significant interactions between light exposure and lens defocus were found (Fig. 2 *C* and *D* and *SI Appendix*, Tables S3 and S4). Neither red light nor green light produced any significant suppression of either the refractive change or AL compared with WL only (Fig. 2 *C* and *D*, red bars). In contrast, blue light produced a modest suppression of refractive change (Fig. 2*C*, blue bar) and a change in AL (Fig. 2*D*, blue bar) that was closer to the week 0 control compared with the WL cohort (Fig. 2*D*, black and white bar). VL produced the most robust response and significantly suppressed refractive change compared with all other wavelengths (Fig. 2*C*, purple bar) and produced an AL that was indistinguishable from the week 0 control with WL (Fig. 2*D*, purple bar). Thus, among multiple wavelengths of visible light, only VL was shown to suppress myopia progression in both refraction and AL in LIM mice.

### OPN5 Is Required for VL Suppression of Myopia.

**Fig. 3.**
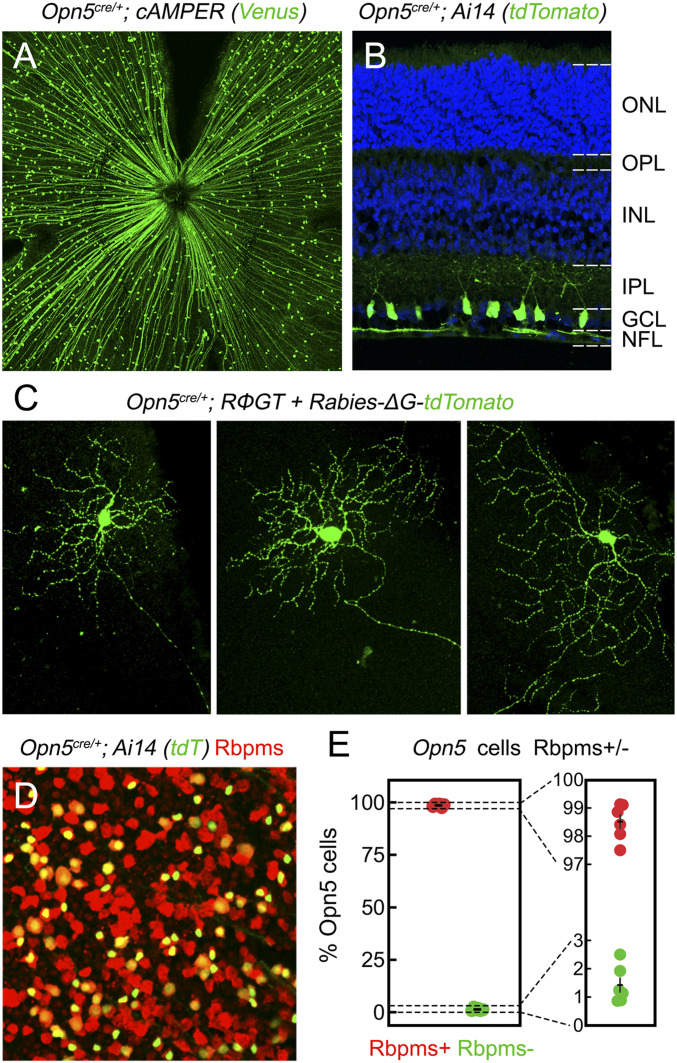
Opn5 expression is limited to RGCs. (*A*) Retinal localization of reporter+ cells (green) in en face retina from cre-dependent *cAMPer* (*A*) or in sections from the Ai14 (*B*) mouse lines crossed to *Opn5*^*cre*^. NFL, nerve fiber layer; GCL, ganglion cell layer; IPL, inner plexiform layer; INL, inner nuclear layer; OPL, outer plexiform layer; and ONL, outer nuclear layer. (*C*) Morphologies of *Opn5* cells assessed through intravitreal injection of modified rabies virus (Rabies-∆G-tdTomato) in *Opn5*^*cre*^*; RΦGT* mice. (*D* and *E*) Detection and quantification of cells in the *Opn5*^*cre*^*; Ai14* line (*n* = 6 mice) that express the RGC-specific marker, Rbpms (*D*, in red), represented as a proportion of all reporter+ cells (*E*). In *E*, an expanded set of axes is shown on the right.

Neuropsin (OPN5) was discovered in 2003 (34). It is required for direct photoentrainment of the mouse retinal and corneal circadian clocks (35). In addition, a VL–OPN5 pathway regulates eye vascular development via dopamine (36), a neuromodulator implicated in myopia (15). The retinal cells that express *Opn5* can be identified by combining the *Opn5*^*cre*^ allele (37) with different cre reporters. When *Opn5*^*cre*^ is combined with the cAMPER allele, cre activity is reported by venus fluorescent protein and is observed in scattered cell bodies as well as radial axon bundles indicative of RGCs (Fig. 3*A*). Consistent with this, cryosections from *Opn5*^*cre*^*; Ai14* mice show positive cell bodies within the ganglion cell layer (Fig. 3*B*, GCL) and axon bundles within the nerve fiber layer (Fig. 3*B*, NFL). When a ΔG rabies virus (38) was used to label *Opn5*^*cre*^-expressing cells sparsely, we identified axons and dendritic arborization patterns typical of RGCs (Fig. 3*C*). Rbpms is an established pan-RGC marker (39) and when applied to *Opn5*^*cre*^*; Ai14* retina, (Fig. 3*D*) shows complete overlap with *Opn5*-lineage cells (Fig. 3*E*). These and prior analyses (35, 37) indicate that *Opn5* expression in the retina is restricted to a subset of RGCs.

**Fig. 4.**
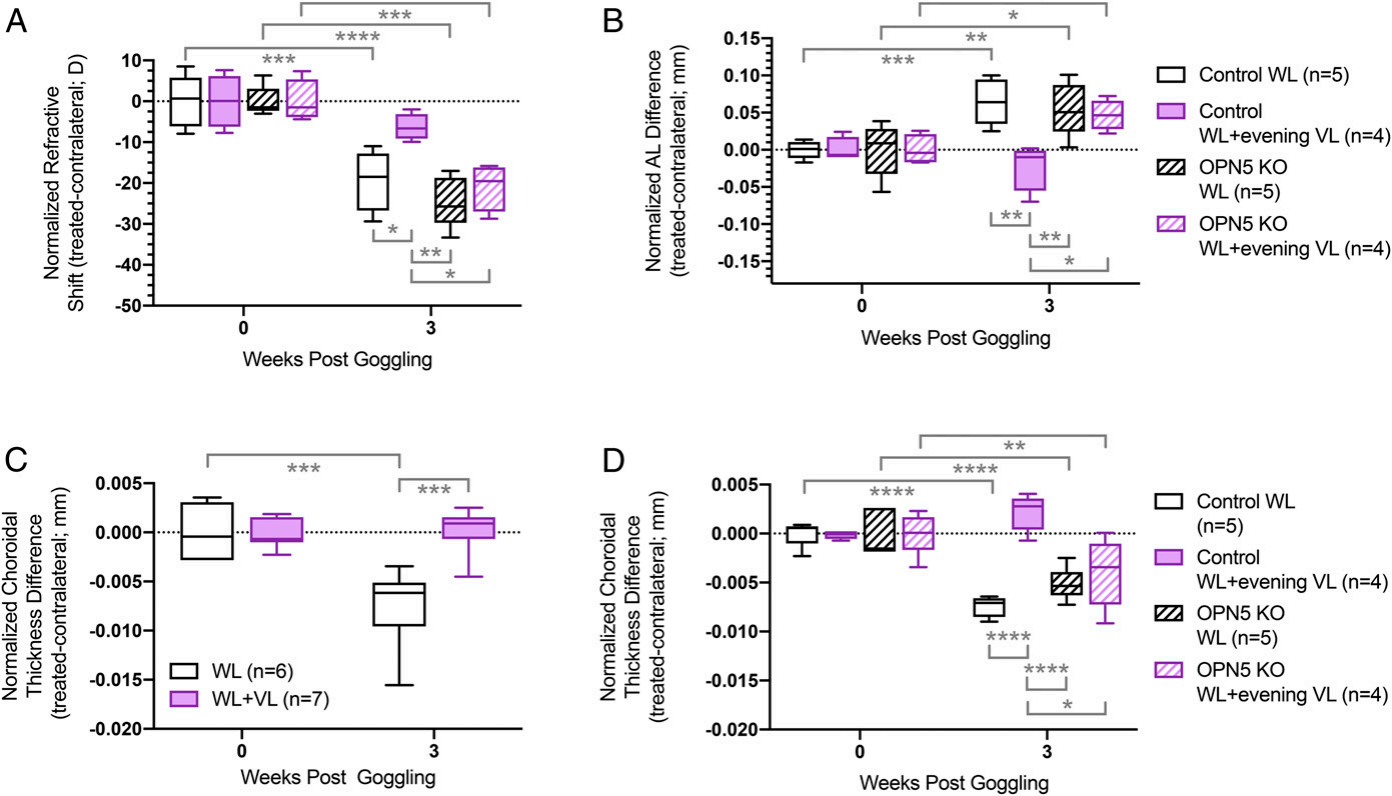
The protective effects of VL in LIM requires OPN5. (*A*–*D*) Measurements of refractive shift (*A*), AL (*B*), and choroidal thickness (*C* and *D*) in the eyes of mice subjected to the lens-induced myopia protocol. The data are displayed as box plots showing the minimum and maximum (error bars), the interquartile range (box), and the median value (bold horizontal line within the box). In *A*, *B*, and *D*, cohorts of *Opn5*^*fl/fl*^ control (white and violet solid shaded bars) and *Chx10-Cre; Opn5*^*fl/fl*^ experimental mice (black and violet cross-hatched bars) were assessed. These assays were performed either in standard WL (white bars and black cross-hatched bars) or in WL + evening VL (violet bars and violet cross-hatched bars). In *C*, all mice were wild type of the C57BL/6J background. The data shown is the difference between the treated and contralateral eyes normalized to baseline. *P < 0.05, **P < 0.01, ***P < 0.001, ****P < 0.0001. The values and statics for *A*–*D* are shown in *SI Appendix*, Tables S5–S8, respectively.

Mouse OPN5 has a λ_max_ of 380 nm, exactly the peak wavelength of VL (40). Thus, we considered the possibility that the VL suppression of myopia was dependent on OPN5. To test this hypothesis, we crossed the *Opn5*^*fl*^ mouse line (a *loxP*-flanked conditional allele) (36) with the *Chx10-Cre* mouse line (41) to generate *Opn5*^*fl/fl*^ control and *Chx10-Cre; Opn5*^*fl/fl*^ experimental mice in which *Opn5* was deleted specifically in the retina. We then used cohorts of these mice in the VL LIM suppression assay (Fig. 4 *A* and *B* and *SI Appendix*, Tables S5 and S6). In normal lighting without VL, both *Opn5*^*fl/fl*^ (control, *n* = 5) and *Chx10-Cre; Opn5*^*fl/fl*^ (experimental, *n* = 5) mice showed a significant refractive shift (Fig. 4*A*, white bar, black cross-hatched bar) and axial lengthening (Fig. 4*B*, white bar, black cross-hatched bar) that were statistically indistinguishable. Importantly, this shows that *Opn5*-conditional null mice possess a fully responsive emmetropization pathway.

When cohorts of *Opn5*^*fl/fl*^ control and *Chx10-Cre; Opn5*^*fl/fl*^ experimental mice were subject to the LIM protocol with WL + evening VL, the response of the two genotypes was distinct. *Chx10-Cre; Opn5*^*fl/fl*^–conditional null mice showed a refractive shift (Fig. 4*A*, violet cross-hatched bar) and an AL change (Fig. 4*B*, violet cross-hatched bar) indistinguishable from the WL control mice of either genotype. By contrast, control mice (*Opn5*^*fl/fl*^) showed a complete suppression of both the refractive shift (Fig. 4*A*, violet bar) and axial lengthening (Fig. 4*B*, violet bar). These data show that induced myopia can be suppressed by VL in an OPN5-dependent manner. This is consistent with the function of OPN5 as a VL-sensitive opsin.

### VL Regulates Choroidal Thickness in an OPN5-Dependent Manner.

Choroidal thickness is known to be reduced in myopic eyes in both the human and in animal models (32). This change is believed to be partly responsible for the regulation of AL and refractive performance. To determine whether choroidal thickness was regulated by VL and by OPN5, we performed two experiments (Fig. 4 *C* and *D* and *SI Appendix*, Tables S7 and S8). In the first, we measured choroidal thickness in wild-type mice using the LIM assay comparing standard WL (Fig. 4*C*, WL) with WL + evening VL (Fig. 4*C*, WL + VL). This showed that under WL, LIM resulted in a significantly thinner choroid (Fig. 4*C*, white bars). By contrast, the addition of evening VL completely suppressed choroidal thinning.

In a second experiment, we measured choroidal thickness in cohorts of *Opn5*^*fl/fl*^ control and *Chx10-Cre; Opn5*^*fl/fl*^ experimental mice subject to the LIM protocol with WL with or without evening VL (Fig. 4*D*). As expected, under WL, the choroid of wild-type mice thinned significantly under the LIM protocol (Fig. 4*D*, white bars). This was also true for the *Opn5*-conditional null (Fig. 4*D*, black hatched bar), again showing that this mutant is capable of a response. When control mice were subject to the LIM protocol under WL + evening VL, the choroidal thinning response was completely suppressed (Fig. 4*D*, violet bars). Notably, under this same protocol, *Opn5*-conditional mutant mice showed choroidal thinning that was significantly different from wild-type mice (Fig. 4*D*, compare violet cross hatched bar with violet bar). These data show that choroidal thickness is regulated by VL and that *Opn5*-expressing RGCs are crucial for this response.

## Discussion

VL has been reported to inhibit myopic development in mice undergoing lens defocus (27). In the present study, we additionally show that VL suppression of myopia is dependent on the time of day at which mice were exposed to VL. Furthermore, this effect was eliminated in mice with *Chx10-cre*–mediated (41) retina-specific conditional deletion of *Opn5*. Since *Opn5* is known to be VL sensitive and expressed in a population of RGCs (35, 36), this analysis suggests that VL activation of *Opn5* in RGCs regulates growth of the eyeball under hyperopic stimulation.

OPN5 was recently discovered as a nonvisual opsin in mammals as well as other vertebrates including the chick and zebrafish (34, 42, 43). OPN5-related opsins fall into three subgroups according to their position on a molecular dendrogram: OPN5m, OPN5-like 1 (OPN5L1), and OPN5-like 2 (OPN5L2). Mammals including humans and mice only contain Opn5m, while chick and zebrafish have all three subtypes (43). OPN5 may share the same function across species, and VL has been shown to regulate several biological functions through this opsin (44, 45). Recently, OPN5 has been shown to be necessary and sufficient for photoentrainment of the local circadian clock of the mouse retina (35) but also appears to make a contribution to photoentrainment of the locomotor activity cycle that is regulated by the central clock of the suprachiasmatic nucleus (SCN) (46). OPN5 was also found to mediate light-dependent vascular development in the mouse eye through regulation of the reuptake of dopamine, a neuromodulator that also has antivascular activity (36). Dopamine is known to be an important regulator of refractive homeostasis (15). OPN5 also mediates direct light responses of hypothalamic neurons (37) and in melanoblasts of the skin (47). Importantly, in the latter analysis, the 380-nm λmax sensitivity of OPN5 was confirmed with an action spectrum for circadian clock entrainment. In the current study, we have shown that OPN5 has a crucial role in mediating the effect of VL on regulating growth of the eyeball in response to hyperopic defocus.

Considering the known functions of OPN5, it is possible that the action of VL in suppressing myopia involves the retinal circadian clock. The local circadian rhythm plays an important role in maintaining retinal functions such as the metabolism of outer-segment disk membranes of photoreceptors (48), the light sensitivity regulation in day and night (49), and visual information processing (50). Direct evidence that the retinal circadian clock is involved in normal refractive development of the eye comes from the demonstration that mutant mice in which the clock gene *Bmal1* has been conditionally deleted from the retina show a myopic shift (51, 52). We hypothesize that a VL–OPN5–retinal circadian clock pathway is required for normal refractive development of the eye. We further suggest that myopia progression could be suppressed when VL is delivered near the end of the light cycle (17:00 to 20:00 and 20:00 to 23:00 in our experiments) in mice. Since mice are nocturnal animals, this timing of VL exposure may be equivalent to dawn exposure for humans. One hypothesis is that VL may suppress myopia progression by eliciting a robust retinal circadian clock rhythm.

This “VL–OPN5–retinal circadian clock hypothesis” may in part explain the myopia boom observed in recent decades: In natural sunlight, VL is always present and is, of course, delivered with the rhythm of the normal light–dark cycle (10). However, human OPN5 is likely to be understimulated in modern society because almost no VL (less than 400 nm) is produced by artificial light sources. Furthermore, UV-protective coatings on windows is standard and eliminates much of the VL that might be available from daytime lighting (9, 10). This has created a situation in which the human retina rarely receives any cue for adjusting its own circadian rhythm. Together with the arrhythmic availability of blue light, this may result in deregulated growth of the eyeball and susceptibility to myopia.

Our data also support the suggestion that a VL–OPN5–retinal pathway regulates choroidal thickness. The choroid likely changes its thickness as part of the emmetropization response and is found to be thinner in myopic eyes than normal eyes (31, 33, 53). Furthermore, the choroid changes its thickness with a diurnal pattern (54), suggesting that this parameter may also be circadian clock controlled. Myopia induction in animal models caused decreased choroidal thickness, and this is likely to be the trigger for reshaping the eyeball (30, 32, 55, 56). Our data show that VL exposure diminished the decrease of choroidal thickness caused by hyperopic defocus, and the effect was also dependent on retinal OPN5. Considering the contribution of other nonvisual photoreceptors in maintaining the homeostasis of organs in individuals (57–61), it is possible that VL/OPN5 pathway plays a role in protecting the eyeball from overreacting to defocus by controlling the thickness of the choroid. The mechanism of OPN5-dependent regulation of choroidal thickness needs further investigation. OPN5 plays an important role mediating vascular development in mouse eye before P8 (36). Since the mice we used in our experiment were much older (P21 to P42), the mechanism for changing the thickness of the choroid is likely to be distinct.

We also found a partial protective effect of blue light in LIM mice in our study, and this is similar to reports in guinea pigs and chicks (18–22). Interestingly, blue light may have no effect or the opposite effect on tree shrews and rhesus monkeys (23, 62). One possible explanation is that longitudinal chromatic aberration (LCA) may guide the eye to achieve emmetropization (21, 27, 54, 62). As argued in Gawne et al., LCA might be treated as either a “target” or a “cue” in the retina of different species and thus lead to distinct responses (23). In both cases, it is proposed that the blue light–responsive photoreceptors are rods or S-cones (54).

We conjecture that VL controls eye growth using a mechanism distinct from that of blue light. While VL can be detected partially by blue cones in human retina, UV cones in mouse, and violet-sensitive cones in the chick (40, 63, 64), in this report we found that the protective effect of VL was completely eliminated in OPN5-conditional null mice. This indicates that VL regulates eye growth via OPN5 but not via a visual opsin. This further indicates that, consistent with prior studies (36), sufficient VL can reach mouse retina to stimulate OPN5.

OPN5 in human retina has almost the same absorption spectrum as mouse OPN5 and thus suggests great potential for using VL as an option for preventing myopia. Additionally, blue light is not suitable for preventing myopia because it would stimulate intrinsically photosensitive RGCs (ipRGCs), causing unpredictable influences on the SCN circadian clock (40, 65–67). The spectrum of the VL source we used in our study is very narrow and emitted almost no light that would stimulate ipRGCs (40). Together with the fact that the VL effect was eliminated in OPN5-conditional null mice, we suggest that ipRCGs are unlikely to play an important role in VL–OPN5 pathway.

Humans are insensitive to UV light because it is absorbed by the cornea and lens (68). By contrast, VL can reach the human retina and is defined as a part of the visible spectrum according to the International Commission on Illumination and the Comité International des Poids et Mesures (26). The lighting environment of modern society can be extremely unnatural: We may be suffering from the hazard of arrhythmic blue light but also from VL deprivation. The sudden absence of VL that is a consequence of indoor living may result in aberrant regulation of the retinal circadian clock and may promote the myopia boom (4). Interestingly, the retinal circadian clock might also be the downstream of two other pharmacologically well-studied pathways that are involved in myopia: acetylcholine signaling through muscarinic and/or nicotinic acetylcholine receptors and dopamine pharmacology (69). We believe that VL–OPN5 pathway could be a practical intervention targeting myopia progression worldwide.

## Materials and Methods

### Mice.

All procedures were approved by the Ethics Committee on Animal Research of the Keio University School of Medicine adhered to the Association for Research in Vision and Ophthalmology Statement for the Use of Animals in Ophthalmic and Vision Research, the Institutional Guidelines on Animal Experimentation at Keio University, and the Animal Research: Reporting of In Vivo Experiments guidelines for the use of animals in research. All wild-type C57BL/6J mice were obtained from CLEA Japan, Inc. *Chx10-Cre* mice line under the C57BL/6J genetic background obtained from The Jackson Laboratory crossed with *Opn5*^*fl/fl*^ mice line under the 129/SvJ1 genetic background to generate OPN5 conditionally knockout mice (*Chx10-Cre; Opn5*^*fl/fl*^). *Opn5*^*fl/fl*^ without *Chx10-Cre* littermate mice were used as control. All mice were fed with normal chew and water ad libitum. Three to four mice with the head-mounted frame were kept in one cage with ∼50-lx background fluorescent lamp light (color temperature: 5,000 K) for 12 h from 08:00 to 20:00 in all the experiments. All the mice used in VL exposure experiments at different times of day, wavelength specificity experiments, and the experiments investigating the involvement of OPN5 underwent lens-induced myopia starting at 3 wk of age for 3 wk.

### Intravitreal Viral Delivery and Opn5-RGC Labeling.

Opn5cre; RΦGT mice (*n* = 2) were anesthetized with ventilated isoflurane (4% induction) and maintained at 1 to 2% for the remainder of the procedure using a rebreather. Mice were subsequently placed under a dissecting microscope, had proparacaine drops (0.5% USP, Sandoz) applied to their right eye, and a pilot incision was made at the limbus with a sterile gauge 271/2 needle. A Hamilton syringe was then inserted into the pilot incision with the needle positioned in the vitreous cavity, and 1 µL of CVS-N2cΔG/EnvA-tdTomato (referred to as Rabies-∆G-tdTomato) was injected at a titer of 1.0 × 10^9^ plaque-forming units/mL. An additional 30 s elapsed after all contents of the syringe were injected to prevent backflow or leakage. Following the injection, mice were observed until recovery from the anesthetic and returned to their home cage. Approximately 3 to 4 wk following intravitreal injections, mice were euthanized, and the eye was processed for immunohistochemistry as described.

### Tissue Processing and Immunohistochemistry.

Mice were deeply anesthetized via isoflurane inhalation and euthanized via cervical dislocation. Eyes were subsequently removed and fixed in 4% paraformaldehyde for 45 min at room temperature. After fixation, the eyes were rinsed twice with phosphate-buffered saline (PBS) and stored in fresh PBS at 4 °C until further processing. For whole mounts (Fig. 3 *A*, *C*, and *D*), retinae were dissected out of the eye and four cuts were made to aid in mounting. Explanted retinae were washed twice in PBS for 15 min followed by an incubation in 0.5% PBS + Triton-X (PBST) for 45 min. Retinae were then subjected to mild antigen retrieval (50% acetone in water) for 15 min before blocking in 10% normal donkey serum in 0.5% PBST (blocking buffer) for 1 h. Primary antibodies were applied in blocking buffer for 3 d at 4 °C, washed six times in PBS (15 min per wash), and incubated in secondary antibodies (1:1,000) + Hoechst (1:10,000) for 2 d at 4 °C. On the final day, retinae were washed six times in PBS (30 min per wash) and mounted in Fluoro-Gel (Electron Microscopy Sciences). The following primaries were used in whole mount retinal analysis: chicken anti-green fluorescent protein (1:1,000; Abcam ab13970), rabbit anti-dsRed (1:1,000; Takara Cat. no. 632496), rabbit anti-Rbpms (1:300; Abcam ab152101), rabbit anti-Foxp2 (1:300; Abcam ab16046), mouse anti-Brnc3 (1:200; Santa Cruz Biotechnology sc-81980), mouse anti-Calretinin (1:300; Milipore, MAB1568), and Isolectin-647 (1:1,000; ThermoFisher Scientific, Cat. no. I21411). For retinal sections (Fig. 3*B*), retinae were subjected to similar processing as whole mounts with minor changes. Retinae were explanted from the fixed eye and cryoprotected in 15% sucrose for 15 min, followed by 30% sucrose overnight. Retinae were mounted in optimal cutting temperature (OCT) media and snap frozen on dry ice, sectioned at 10 µm, and incubated in Hoechst (1:10,000) for 5 min before mounting and coverslipping. All imaging was performed on a Nikon A1 inverted microscope.

### LIM in Mice.

LIM was induced in mice according to previous reports (28, 29). Briefly, mice were put into general anesthesia by a combination of midazolam (Sandoz K.K.), medetomidine (Domitor, Orion Corporation), and butorphanol tartrate (Meiji Seika Pharma Co., Ltd.), MMB for short. The scalp was cut to expose an ∼0.8-cm^2^ area of the skull, and the periosteum was removed with etching fluid. Then, a set of eyeglasses were attached to the mouse’s head using a self-cure dental adhesive system (Super-Bond, SUN MEDICAL). The eyeglasses were designed specifically for mice and forged by a three-dimensional printer. The eyeglasses have a joint part that allow the position of left and right frame to be adjusted according to the shape of the mouse skull or be taken off for cleaning. The lenses on the eyeglasses were customized from human hard contact lens by a manufactory in Japan. All the left sides of eyeglasses used in this paper were attached with 0-D lenses as internal control, and right sides of the eyeglasses were attached with −30-D lenses. The eyeglasses were removed for cleaning at least twice a week for each mouse.

### Refraction, AL, and Choroid Thickness Measurements.

Refractions and ALs were measured according to previous reports (28, 29). Briefly, an infrared photorefractor (Steinbeis Transfer Center) was used to measure the refractive state. Tropicamide and phenylephrine hydrochloride solution (Mydrin-P ophthalmic solution, Santen Pharmaceutical) were applied to the mouse eye 5 min before the measurement to ensure mydriasis and cycloplegia. Refractions were taken along the optic axis under general anesthesia induced by MMB. After the measurement of refraction, the AL and choroid thickness were analyzed by a SD-OCT system (Envisu R4310, Leica) tuned for mice. The AL was defined as the distance from the corneal vertex to the RPE layer near the optic nerve (Fig. 1*B*). The choroid thickness was measured with the OCT system according to a previous report (32). Briefly, the area of the circumference at 0.5 mm from the disk circled at the border of the RPE, and the posterior surface of the choroid was quantified with ImageJ (NIH). Then, the average choroid thickness was calculated by dividing the area with circumference.

Measurements of refraction, AL, and choroid thickness were performed twice for each mouse: before initiation of LIM (0 W) and 3 wk afterward. The relative differences between eyes were calculated as follows: differences between right eye and left eye (0 or 3 W) minus the average value of differences between right eye and left eye in 0 W in each group, with all values normalized to baseline.

### Light Interventions.

Approximately 50 lx of white background fluorescent lamp light was applied from 08:00 to 20:00 every day (WL) to all the mice with or without different light interventions. Spectral irradiance of the light environment was measured by the Blue-Wave spectrometer UVNb-50 (StellerNet). For VL exposure at different times of day, beside the white background light, 400 μw/cm2 (360 to 400 nm) of VL was added at different times of day for 3 wk: 05:00 to 08:00 (WL + predawn VL), 08:00 to 20:00 (WL + daytime VL), 00:00 to 24:00 (WL + continuous VL), 05:00 to 08:00 (WL + morning VL), 17:00 to 20:00 (WL + evening VL), and 20:00 to 23:00 (WL + postdusk VL). For wavelength specificity experiments, 400 μw/cm^2^ of VL (360 to 400 nm), blue light (440 to 480 nm), green light (500 to 540 nm), or red light (610 to 650 nm) irradiation was added from 17:00 to 20:00 every day, respectively, in each group for 1 wk. The control group was exposed to the background light only. For the experiment investigating the involvement of OPN5, 400 μw/cm^2^ (360 to 400 nm) of VL was added from 17:00 to 20:00 (evening VL) every day for 3 wk. The light source of violet, blue, green, and red light were LEDs made by NICHIA Japan. The experimental conditions are summarized in Table 1.

## Statistical Analyses.

Two-way repeated or nonrepeated ANOVA with Sidak’s or Tukey’s multiple comparison tests were used to analyze statistical significances of all the data in this paper (Graphpad Prism 8.0). *P* < 0.05 was considered significant. All data are presented as mean ± SEM. All values and statistics for charts are summarized in *SI Appendix*, Tables S1–S8.

## Supplementary Material

Supplementary File

## Data Availability

All the source data are included in the article and/or supporting information. Source data are also publicly available at Figshare (DOI: 10.6084/m9.figshare.14089970).
