# Supplementary material for: Violet light suppresses lens-induced myopia via neuropsin (OPN5) in mice
Source: Proc Natl Acad Sci U S A. 2021 May 24;118(22):e2018840118. doi: 10.1073/pnas.2018840118 (PMC8179197; doi:10.1073/pnas.2018840118)
Supplement: Supplementary File [file pnas.2018840118.sapp.pdf]

PNAS
www.pnas.org

Supplementary Information for

Violet light suppresses lens-induced myopia via neuropsin (OPN5) in mice

Xiaoyan Jiang[1,2†], Machelle T. Pardue[3,4†], Kiwako Mori[1,2], Shin-ichi Ikeda[1,2], Hidemasa Torii[1,2], Shane D'Souza[5], Richard A. Lang[5,6], Toshihide Kurihara[1,2*], Kazuo Tsubota[1,7*]

[1]Department of Ophthalmology, Keio University School of Medicine, Shinjuku-ku, Tokyo 160-8582, Japan
[2]Laboratory of Photobiology, Keio University School of Medicine, Shinjuku-ku, Tokyo 160-8582, Japan
[3]Center for Visual and Neurocognitive Rehabilitation, Atlanta VA Healthcare System, Atlanta, GA 30033, United States
[4]Biomedical Engineering, Georgia Institute of Technology and Emory University, Atlanta, GA 30322, United States
[5]Visual Systems Group, Abrahamson Pediatric Eye Institute, Division of Pediatric Ophthalmology, Cincinnati Children's Hospital Medical Center, Cincinnati, OH 45229, United States
[6]Department of Ophthalmology, College of Medicine, University of Cincinnati, Cincinnati, OH 45221, United States
[7]Tsubota Laboratory, Inc., Tokyo 160-8582, Japan

†These authors are contributed equally to the work and request the double first authorship

*Corresponding authors:

Toshihide Kurihara, MD, PhD
Laboratory of Photobiology, Department of Ophthalmology, Keio University School of Medicine; 35 Shinanomachi, Shinjuku-ku, Tokyo 160-8582, Japan
Tel: +81-3-5363-3204, Fax: +81-3- 5363-3274
E-mail: kurihara@z8.keio.jp

Kazuo Tsubota, MD, PhD
Department of Ophthalmology, Keio University School of Medicine; 35 Shinanomachi, Shinjuku-ku, Tokyo 160-8582, Japan.
Tel: +81-3-5363-3269, Fax: +81-3- 3358-5961
E-mail: tsubota@z3.keio.jp

This PDF file includes:

Table S1 to S8

Table S1. Numeric data for refractive shift: Figure 1D

Two-way repeated measures ANOVA, interaction time x treatment: $F(5, 36) = 4.75$, $p=0.002$									
**Experimental Cohort**	**Weeks of lens induced myopia**		**p-values: comparison between**						
	0	3	0 and 3 weeks	different lighting conditions at 3 weeks					
	min-P_{25}/median/ P_{75}-max	min-P_{25}/median/ P_{75}-max							
**WL**	-1.960/-0.740/0.860	-28.330/-23.170/-20.500	**<0.0001**	with					
**WL + predawn VL**	-7.357/-0.437/7.263	-37.517/-28.027/-20.897	**<0.0001**	>0.9999	with				
**WL + daytime VL**	-3.923/-0.893/3.632	-27.276/-24.288/-16.836	**<0.0001**	>0.9999	0.9969	with			
**WL + continuous VL**	-3.015/0.140/4.475	-23.748/-13.490/-8.468	**0.0027**	0.2681	0.1123	0.7939	with		
**WL + evening VL**	-6.254/2.599/4.516	-18.864/-10.481/-3.786	**0.0195**	**0.0058**	**0.0026**	0.1044	0.9976	with	
**WL + post dusk VL**	-10.965/2.340/-10.965	-24.445/-10.010/10.195	0.4461	**0.0137**	**0.0057**	0.1055	0.9707	>0.9999	
**WL: White light. VL: violet light. P_{25}: 25th percentile. P_{75}: 75th percentile.**									

Table S2. Numeric data for axial length difference: Figure 1E

Two-way repeated measures ANOVA, interaction time x treatment: F(5, 33) = 3.883, p=0.0071									
Experimental Cohort	Weeks of lens induced myopia		p-values: comparison between:						
	0	3	0 and 3 weeks	different lighting conditions at 3 weeks					
	min-25th/median/75th-max	min-25th/median/75th-max							
**WL**	-0.027/0.003/0.018	0.033/0.055/0.082	**<0.0001**	with					
**WL + predawn VL**	-0.013/0.002/0.008	0.037/0.042/0.082	**<0.0001**	>0.9999	with				
**WL + daytime VL**	-0.007/0.0002/0.007	0.031/0.0479/0.071	**0.0025**	>0.9999	>0.9999	with			
**WL + continuous VL**	-0.034/0.0005/0.033	-0.005/0.0127/0.025	0.9349	**0.0165**	**0.0078**	0.1311	with		
**WL + evening VL**	-0.016/0.005/0.011	0.014/0.019/0.033	0.5702	**0.0451**	**0.0208**	0.2822	>0.9999	with	
**WL + post dusk VL**	-0.016/-0.010/0.025	-0.004/0.017/0.042	0.7633	0.2198	>0.9999	0.5637	>0.9999	>0.9999	
**WL: White light. VL: violet light. P_{25}: 25th percentile. P_{75}: 75th percentile.**									

Table S3. Numeric data for refractive shift: Figure 2C

Two-way repeated measures ANOVA, interaction time x treatment: F(4, 35) = 7.596, p=0.0002							
**Experimental Cohort**	**Weeks of lens induced myopia**		**p-values: comparison between:**				
	0	3	0 and 3 weeks	different lighting conditions at 3 weeks			
	min-25th/median/75th-max	min-25th/median/75th-max					
**WL**	-1.814/-0.085/2.861	-31.818/-29.360/-18.368	**<0.0001**	with			
**WL + RL**	-2.188/0.626/4.671	-38.520/-27.019/-20.392	**<0.0001**	0.8717	with		
**WL + GL**	-3.210/-0.248/2.868	-30.596/-24.003/-15.830	**<0.0001**	0.9200	0.3864	with	
**WL + BL**	-4.278/0.178/3.480	-22.628/-17.883/-15.520	**<0.0001**	0.2667	**0.0312**	0.7570	with
**WL + VL**	-3.908/0.345/3.588	-17.095/-6.845/-4.025	**0.0082**	**<0.0001**	**<0.0001**	**0.0004**	**0.0175**
**WL: White light. RL: Red light. GL: Green light. BL: Blue light. VL: violet light. P_{25}: 25th percentile. P_{75}: 75th percentile.**							

Table S4. Numeric data for axial length difference: Figure 2D

**Two-way repeated measures ANOVA, interaction time x treatment: F(4, 35) = 5.723, p=0.0012**								
**Experimental Cohort**	**Weeks of lens induced myopia**		**p-values: comparison between:**					
	0	3	0 and 3 weeks	different lighting conditions at 3 weeks				
	min-25th-median-75th-max	min-25th-median-75th-max						
**WL**	-0.030/0.005/0.20	0.020/0.045/0.080	**<0.0001**	with				
**WL + RL**	-0.012/0.00007/0.013	0.012/0.04067/0.060	**<0.0001**	0.9393	with			
**WL + GL**	-0.005/-0.001/0.009	0.031/0.040/0.060	**<0.0001**	0.9897	0.9983	with		
**WL + BL**	-0.012/-0.002/0.006	0.007/0.014/0.037	**0.0287**	0.2191	0.6528	0.4658	with	
**WL + VL**	-0.032/0.002/0.028	-0.014/0.006/0.010	0.9996	**0.0020**	**0.0211**	**0.0090**	0.4040	
**WL: White light. RL: Red light. GL: Green light. BL: Blue light. VL: violet light. P_{25}: 25th percentile. P_{75}: 75th percentile.**								

Table S5. Numeric data for refractive shift: Figure 4A

**Three-way repeated measures ANOVA, two way interaction time x genotype: F(1, 14) = 10.13, p=0.0067**						
**Experimental Cohort**	**Weeks of lens induced myopia**		**p-values: comparison between:**			
	0	3	0 and 3 weeks	different cohorts at 3 weeks		
	min-25th-median-75th-max	min-25th-median-75th-max				
**Control WL**	-6.105/0.670/5.770	-26.750/-18.500/-12.800	**<0.0001**	with		
**Control WL + evening VL**	-6.236/0.057/6.178	-9.120/-6.600/-3.105	0.4371	**0.0509**	with	
***Opn5* KO WL**	-2.333/-1.498/3.082	-29.740/-25.720/-18.725	**<0.0001**	0.9579	**0.0016**	with
***Opn5* KO + evening VL**	-3.885/-1.498/5.383	-26.970/-19.525/-16.190	**<0.0001**	>0.9999	**0.0140**	>0.9999
**WL: White light. VL: violet light. P_{25}: 25th percentile. P_{75}: 75th percentile.**						

Table S6. Numeric data for axial length difference: Figure 4B

Table S6: Numeric data for axial length difference: Figure 4B							
**Three-way repeated measures ANOVA, three way interaction time x genotype x light: F(1, 14) = 7.657, p=0.0151**							
**Experimental Cohort**	**Weeks of lens induced myopia**		**p-values: comparison between:**				
	0	3	0 and 3 weeks	different cohorts at 3 weeks			
	min-P_{25}-median- P_{75}-max	min-P_{25}-median- P_{75}-max					
**Control WL**	-0.011/0.001.0.010	0.035/0.064/0.095	**0.0008**	with			
**Control WL + evening VL**	-0.010/-0.007/0.017	-0.055/-0.010/-0.001	0.4751	**0.0018**	with		
***Opn5* KO WL**	-0.032/0.009/0.028	0.024/0.051/0.087	**0.0033**	>0.9999	**0.0077**	with	
***Opn5* KO + evening VL**	-0.017/-0.004/0.021	0.028/0.046/0.066	**0.0235**	>0.9999	**0.0119**	>0.9999	
**WL: White light. VL: violet light. P_{25}: 25th percentile. P_{75}: 75th percentile.**							

Table S7. Numeric data for choroidal thickness difference: Figure 4C

**Two-way repeated measures ANOVA, interaction time x treatment: F(1, 11) = 16.73, p=0.0018**				
**Experimental Cohort**	**Weeks of lens induced myopia**		**p-values: comparison between:**	
	0	3	0 and 3 weeks	different cohorts at 3 weeks
	min-P_{25}-median- P_{75}-max	min-P_{25}-median- P_{75}-max		
**WL**	-0.003/-0.0004/0.003	-0.010/-0.006/-0.005	**0.0004**	with
**WL + evening VL**	-0.0010/-0.001/0.002	-0.0007/0.001/0.002	0.9968	**0.0002**
**WL: White light. VL: violet light. P_{25}: 25th percentile. P_{75}: 75th percentile.**				

Table S8. Numeric data for choroidal thickness difference: Figure 4D

**Three-way repeated measures ANOVA, interaction time x genotype x light: F(1, 16) = 9.562, p=0.0070**						
**Experimental Cohort**	**Weeks of lens induced myopia**		**p-values: comparison between:**			
	0	3	0 and 3 weeks	different cohorts at 3 weeks		
	min-P_{25}-median- P_{75}-max	min-P_{25}-median- P_{75}-max				
**Control WL**	-0.001/0.0006/0.0007	-0.009/-0.0071/-0.0066	**<0.0001**	with		
**Control WL + evening VL**	-0.006/-0.00008/0.0002	0.0004/0.003/0.0036	0.0728	**<0.0001**	with	
***Opn5* KO WL**	-0.0018/-0.002/0.003	-0.0063/-0.005/-0.004	**<0.0001**	0.9708	**<0.0001**	with
***Opn5* KO + evening VL**	-0.002/0.00008/0.002	-0.007/-0.00342/-0.001	**0.0012**	0.2420	**0.0119**	>0.9999
**WL: White light. VL: violet light. P_{25}: 25th percentile. P_{75}: 75th percentile.**						
